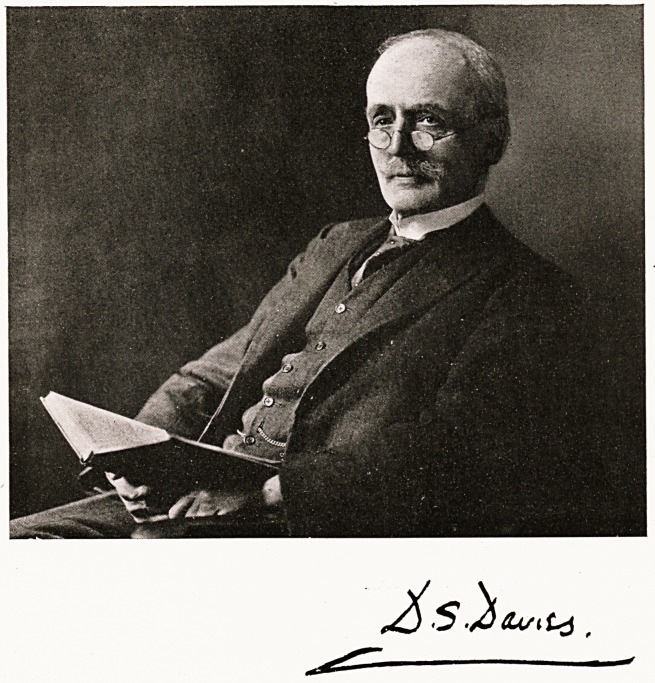# David Samuel Davies

**Published:** 1933

**Authors:** 


### Obituary

### DAVID SAMUEL DAVIES, M.D., LL.D.

*Late Medical Officer of Health, Bristol.*

WE regret to announce the death on 26th March, at Exmouth, of Dr. D. S. Davies, who was for forty-two years Medical Officer of Health of the City and Port of Bristol.

David Samuel Davies was born in Bristol in 1855, and from Bristol Grammar School went to St. Thomas's Hospital, qualifying as M.R.C.S. and L.S.A. in 1879. Two years later, after serving as house-surgeon to St. Thomas's, he became L.R.C.P. and M.B.Lond., and obtained the Cambridge D.P.H. For several years he held office under the Local Government Board as medical inspector on the cholera survey and general sanitary survey of England and Wales, and then in 1886 was appointed M.O.H. for Bristol, in succession to his father, Dr. David Davies. In 1891 he graduated M.D.Lond. in State Medicine, and in 1912 the University of Bristol, for which he lectured and examined in public health, conferred on him its LL.D. During the war he was specialist sanitary adviser to the Southern Command, with the rank of lieutenant-colonel R.A.M.C.(T.), having previously held the rank of surgeon-colonel in the 1st Gloucestershire R.G.A. Volunteers. Dr.

Davies was President of the Bristol Medico-Chirurgical Society in 1900–1. He had held office as President of the Society of Medical Officers of Health in 1906–7 and of the Bath and Bristol Branch of the B.M.A. in the same year.

He married in 1882 Louisa Gertrude Despard, who died in 1929. He married again in 1930 Mary Harriett, widow of the Rev. J. F. E. Faning, who survives him. To her and to his two sons we offer our deep sympathy.

An Appreciation by Sir Arthur Newsholme, K.C.B.

I learnt with much regret of the death of my old friend and fellow-student, Dr. D. S. Davies, who for so many years has been a leader in public health and one of our most distinguished practical epidemiologists.

His life-work was done in Bristol, and that ancient city has cause to be proud of the family of Davies, father and son, who have safeguarded the health of the city for over half a century, who have been largely responsible for the disappearance of typhus (of which disease, imported from Ireland, Bristol was for long an endemic centre), and for practical conquest, achieved during the son's forty years' public health work, over such diseases as typhoid fever and small-pox.

D. S. Davies's investigation of a Clifton outbreak of typhoid fever, due to an infected milk supply, has long been quoted in official writings and text-books as a classic in the methods employed in the investigation and a model in the skill in setting out the results attained. The fullest account of this outbreak is, I believe, contained in the *Journal of Hygiene.* But it was Davies's investigations into local outbreaks of small-pox which, above all, excited the admiration and wonder of his colleagues, who knew the difficulties involved in tracing the source and track of infection. He was, in fact, a veritable sleuth-hound in tracing channels of infection, and in thus limiting outbreaks of small-pox, which but for his superlative skill would have seriously damaged the city he served.

D. S. Davies was a pioneer in the use of the laboratory as an important aid to epidemiological investigation and the prevention of infection. I believe he was one of the earliest, if not the earliest in England, to start a municipal laboratory for the examination of suspected diphtheria smears ; and the account of the Clifton outbreak already mentioned shows how early he utilized the Widal reaction in investigating sources and channels of typhoid infection.

For many years we had seldom met ; but one always felt that D. S. Davies was a loyal friend, a wise counsellor, always regardful of the "other fellow's" view, and a man with a single eye to duty.

### A STUDENT'S MEMORIES.

During my student career in the Bristol Medical School I regarded D. S. Davies as one of the best, probably the best, of all the teachers upon whom I attended. His tremendous enthusiasm for his subject was infectious, and some of the tales he had to tell of the tracking down of epidemics to their source appealed to me as fascinating. It seems worth while to keep his memory green by telling again the story of the epidemic of typhoid fever in Bristol in the autumn of 1897, and his investigations into its origin.

During October-November 208 cases of enteric fever were notified in the town, in eighty-eight different houses. Some Clifton College houses suffered severely; there were twenty-nine cases in one of them. The outbreak was puzzling, because it was too localized to be due to water, and it followed three different milk rounds. There were a few cases at Bower Ashton and Leigh Woods, but the great bulk were in Clifton and Hotwells. Dr. Davies and his inspectors soon found that many of the infected houses in both these districts were supplied with milk from a farm in Long Ashton, and more than half of the houses on that milk round yielded cases at the rate of about two per house. This seemed clear enough, but this only explained 104 out of 208 cases of the disease, and did not account for eight in Bower Ashton and Leigh Woods. Under the pressure of cross-examination, however, the milkman who served these people admitted that he purchased some milk (which he had no right to do) from the man who distributed the supply from the infected farm, who had no right to sell, and did not account to his employer for the transaction. But even so there were sixteen houses and twenty-two patients with enteric fever who had milk from yet a third source, which was peculiar in that of its three milk rounds two were quite clear, and the third was clear up to the corner of a certain street, but in the very next house there were four cases. Strange to say, at this very point the milkman's route crossed that of the man from the Long Ashton farm. Once again cross-examination showed that a clandestine purchase took place, but that the employer was not told of it. Thus all the cases except ten were accounted for, and those might easily have been infected from little retail shops. Further investigation showed that the farm where the infection started did not water the milk, but washed their churns in the sewage-polluted water of a brook; the inmates and cows were healthy. Following along the houses supplied, it was interesting to notice that one institution which scalded its milk escaped entirely, and persons who only took milk in tea did not suffer. In one girls' school five drank boiled milk and did not suffer; fifteen took it unboiled, and twelve got the disease.

Amusing episodes came to light. Rumour in the town fixed on the wrong source, and one family in a panic changed their milk supply from a perfectly pure one to that from the infected farm! Many people, including one doctor, loudly advertised that the whole epidemic was due to smells from the River Avon, and the unfortunate M.O.H., in spite of his magnificent demonstration of the truth, was compelled to placate public opinion by having several barrels of disinfectant poured into the river.

A. R. S.

## Figures and Tables

**Figure f1:**